# Boosting nutritional quality of *Urtica dioica* L. to resist climate change

**DOI:** 10.3389/fpls.2024.1331327

**Published:** 2024-02-15

**Authors:** Nevena Opačić, Sanja Radman, Mia Dujmović, Sanja Fabek Uher, Božidar Benko, Nina Toth, Marko Petek, Lepomir Čoga, Sandra Voća, Jana Šic Žlabur

**Affiliations:** ^1^ Department of Vegetable Crops, University of Zagreb Faculty of Agriculture, Zagreb, Croatia; ^2^ Department of Sustainable Technologies and Renewable Energy Sources, University of Zagreb Faculty of Agriculture, Zagreb, Croatia; ^3^ Department of Plant Nutrition, University of Zagreb Faculty of Agriculture, Zagreb, Croatia

**Keywords:** stinging nettle, floating hydroponics, nitrates, minerals, specialized metabolites, antioxidant capacity

## Abstract

**Introduction:**

More than ever, traditional agricultural practices need a shift towards more resilient, sustainable, modern and adaptable practices that benefit the health of the planet and people. Today's consumers are constantly on the lookout for novel, highly nutritious foods that have a positive impact on their overall health and well-being. Nettle (Urtica dioica L.) is gaining recognition not only as a popular medicinal plant, but also as a desirable green leafy vegetable rich in phytonutrients. As it is difficult and even expensive to control the quality standards of wild-collected plants, the implementation of sustainable cultivation methods, especially hydroponics, with effective greenhouse management could be a possible solution to obtain a standardized product with high nutritional value. Therefore, the aim of this study was to investigate the effects of four nutrient solutions differing in the content of macro- and micronutrients (especially nitrogen, potassium, calcium, magnesium and iron) and two consecutive cuts on the number of leaves, yield, nitrate and mineral content and the content of specialized metabolites of stinging nettle from a floating hydroponic system.

**Methods:**

Nettle plants were cultivated in a hydroponic system using the floating hydroponics technique. The two-factorial experiment was performed with nutrient solution and consecutive cuts as factors.

**Results:**

The highest yield (2.49 kg/m2) was achieved after the 1st cut with plants cultivated in the nutrient solution with higher nutrient concentration. All tested nutrient solutions resulted in high levels of minerals and bioactive compounds in the plant material (ascorbic acid content of 102.30 mg/100 g fw and total phenolics content of 465.92 mg GAE/100 g fw), confirming floating hydroponics as a sustainable approach for cultivating nettle with enhanced nutritional value and antioxidant potential.

**Conclusion:**

It is important to highlight that the nutrient solution with the lowest nutrient composition yielded the highest concentrations of calcium (5.54%) and iron (180.67 mg/kg dw). Furthermore, it exhibited elevated levels of specific phenolic compounds, including caffeoylmaleic acid, ellagic acid, ferulic acid, naringin, and rutin trihydrate. Notably, this solution demonstrated the lowest nitrate content (4225.33 mg/kg fw) in the plant material. Therefore, it can be recommended as a preferable formulation for hydroponic nettle cultivation.

## 1 Introduction

In the face of increasing global challenges, the agricultural sector is at the forefront of a critical transformation. Climate change, population growth, and increasing scarcity of land and water resources create an urgent need to modernize traditional agricultural practices. Agriculture is one of the world’s largest water consumers and also represents the most extensive land use on the planet covering 1.5 billion ha (Garnett et al., 2013; Rufí-Salís et al., 2020; Steensland and Zeigler, 2021). Competition for resources, both water and land, between food production and other uses will intensify in the future (Steensland and Zeigler, 2021). To address these issues and achieve the European Union’s ambitious sustainability goals, modern agriculture must adapt, innovate, and adopt environmentally conscious techniques (European Commission, 2023a; Marek and Tosun, 2023). The European Union has set a course toward a carbon-neutral circular economy that includes a series of strategic plans to usher in a new era of sustainable agricultural practices. Central to this change is the adoption of modern cultivation methods that not only increase productivity but are also compatible with environmental principles. These methods offer the possibility of bridging the gap between the growing demand for food and the constraints imposed by finite resources (Gonnella and Renna, 2021; Heyl et al., 2023; Monisha et al., 2023).

Vegetable cultivation in the open field is increasingly exposed to many risks and is also a major contributor to nutrient losses, air and water pollution, and biodiversity loss. Cultivation is increasingly shifting to greenhouses, where abiotic factors can be controlled to a certain extent and the influence of climate changes on crop production is less evident (Fernández et al., 2018; Savvas and Gruda, 2018; Ashour et al., 2020; Hamza et al., 2022). Greenhouses offer the advantage of enabling crop cultivation in urban areas, even on unsuitable land, addressing the challenges of dwindling arable land availability and a growing population.

Hydroponic production is a turning point and a suitable solution to overcome the above problems, mainly because of its advantages, which range from good management of natural resources to rational water use and prevention of nutrient losses from the rhizosphere (Gonnella and Renna, 2021; Monisha et al., 2023). In a world struggling with environmental problems, hydroponic systems represent a step toward low input, high resource efficiency, and closed-loop production. These systems enable soilless plant growth through precise water and nutrient management. They offer better control of plant nutrition, more efficient use of space, reduced reliance on herbicides and pesticides, and significant savings in fertilizer and water use with the goal of minimizing nitrate uptake, making them suitable for environmentally sensitive areas. The potential of hydroponic agriculture is also underlined by the fact that it helps to solve the problem of nitrate accumulation in green leafy vegetables, a common problem and an important issue in conventional agriculture (Gonnella et al., 2004; Bian et al., 2020; Rufí-Salís et al., 2020).

Nitrate is still considered an anti-nutrient that is potentially harmful to the consumer, and the amounts in foods and especially vegetables are monitored (Brkić et al., 2017). However, some research suggests that the consumption of plant foods containing nitrate and nitrite can have a positive impact on the treatment of some metabolic disorders, heart disease and gastric ulceration (Lundberg et al., 2008; Rocha, 2021) and that it has a positive effect on the central nervous, the musculoskeletal and the cardiovascular system. Rocha (2021) states that preclinical and clinical evidence suggests that a diet rich in nitrates has the potential to halt, reverse or ameliorate the physiological decline associated with aging. Nevertheless, controlling nitrate content in plant foods is crucial to ensure their safety and nutritional value, as about 80% of nitrates in the diet come from the consumption of vegetables (Brkić et al., 2017; Luetic et al., 2023). Nitrate accumulation is very complex and is influenced by several internal and external factors, highlighting the need for careful management throughout the plant’s growth cycle to optimize the balance between nutritional value and potential risks (Qiu et al., 2014).

In hydroponic systems, the plants receive their nutrients via a nutrient solution that can be tailored to the exact needs of the individual species. The nutrient solution is a decisive factor that prevents nutrient antagonisms, ensures optimal plant nutrition and offers the possibility of influencing the content of minerals and certain metabolites by directly regulating the contents of the individual biogenic elements in the solution (Gonnella et al., 2004; Nicola et al., 2006; Trejo-Téllez and Gómez-Merino, 2012). In addition, it is important to know that an increased nitrogen content in the nutrient solution can have a negative impact on the concentration of specialized metabolites (especially polyphenols), which ultimately affects the overall quality of the final product (Radman et al., 2015). Therefore, it is extremely important to find a suitable nutrient solution formulation that provides the plant with sufficient nutrients without negatively affecting the content of bioactive compounds (Niu et al., 2015; Gruda et al., 2018).

Recently, stinging nettle (*Urtica dioica* L.) has been making a big comeback as a leafy green vegetable (the better counterpart to spinach), gaining superfood status because it is rich in minerals and specialized metabolites that are important for human health (Opačić et al., 2022). However, this plant is also a notable example of a nitrate-accumulating species (Radman et al., 2015), making it a great experimental plant. As far as we know, growing nettle for human consumption using hydroponics is still rare and there are no official guidelines for successful agronomic practices for its cultivation. Such a product from controlled agronomic production is currently not available on the market.

The aim of this study was to investigate the effects of four different nutrient solutions in hydroponic cultivation on the number of leaves, yield, mineral composition, nitrate and specialized metabolites concentration of stinging nettle during two consecutive cuts.

## 2 Materials and methods

### 2.1 Plant material and cultivation conditions

The research was conducted in an unheated greenhouse at the Experimental Station of the Department of Vegetable Crops of the Faculty of Agriculture in Zagreb (45.8150° N, 15.9819° E). The nettle plants were cultivated in a hydroponic system using the floating hydroponics technique. The two-factorial experiment, with nutrient solution and consecutive cuts as factors, was set up according to a randomized complete block design with three replicates. Each replicate consisted of two polystyrene plates and there were six plates in each basin.

Nettle seeds (B&T World Seeds, France) were initially sown on March 11, 2021 in polystyrene plates (0.96 × 0.60 m) containing 102 slots (17.0 × 0.5 cm) and filled with granulated inert substrate perlite (Europerl d.o.o., Croatia). With the sowing of 30 seeds per slot, 3060 plants/plate can be achieved, resulting in a total quantity of 5312 plants/m^2^. During the germination phase, the plates were placed in a greenhouse with a temperature of 15 to 20°C and humidity of 60%. As soon as the nettle plants emerged (March 18), the plates were transferred to 3.0 × 1.2 m (878L) basins filled with water.

To evaluate the effects of different nutrient solutions on the quality of nettle plants, four nutrient solutions were prepared with different nutrient levels according to Lorenz and Maynard (1988) (see **Table 1**), which is reflected in the target electrical conductivity (EC) values of each solution.

**Table 1:** Concentration of nutrient solutions for the hydroponic cultivation of nettle.

Solution	NS1	NS2	NS3	NS4
Salts	mg/L			
KNO_3_	250.99	203.43	177.01	583.88
KH_2_PO_4_	142.7	272.12	180.0	261.56
K_2_SO_4_	0	0	343.46	0
Ca(NO_3_)_2×_4H_2_O	501.5	548.7	1063.0	1093.0
MgSO_4_ × 7H_2_O	256.25	494.06	494.06	512.6
FeEDTA 13%	12.8	13.66	17.07	42.68
H_3_BO_3_	1.32	2.64	5.81	1.59
CuSO_4_ × 5H_2_O	0.026	1.32	1.32	0.26
MnSO_4_ × 4H_2_O	0.79	2.38	3.96	6.08
ZnSO_4_ × 7H_2_O	0.11	0.40	1.32	0.45
Na_2_MoO_4 ×_ 2 H_2_O	0.02	0.07	0.19	0
(NH_4_)_6_Mo_7_O_24_x4H_2_O	0	0	0	0.37
EC	1.5	1.7	2.8	3.0
pH	5.8 – 6.2			

EC, electric conductivity.

Since nettle plants have a relatively long germination period during which nutrient requirements are not as pronounced and in order to minimize the excessive use of costly salts for nutrient solution preparation (in light of sustainable farming), the plates were initially positioned on water-filled basins. Subsequently, on April 13, when the nettle roots had developed, the nutrient solution was added. A multiparameter instrument (Hanna Instruments HI98194, Romania) was used to measure several solution quality parameters, including pH and electrical conductivity (mS/cm), temperature (°C), and oxygen content (mg/L) of the solution. In addition, air temperature and relative humidity were measured regularly in the greenhouse using a tabletop thermohygrometer (Agrologistika d.o.o., Croatia).

**Figure 1:**
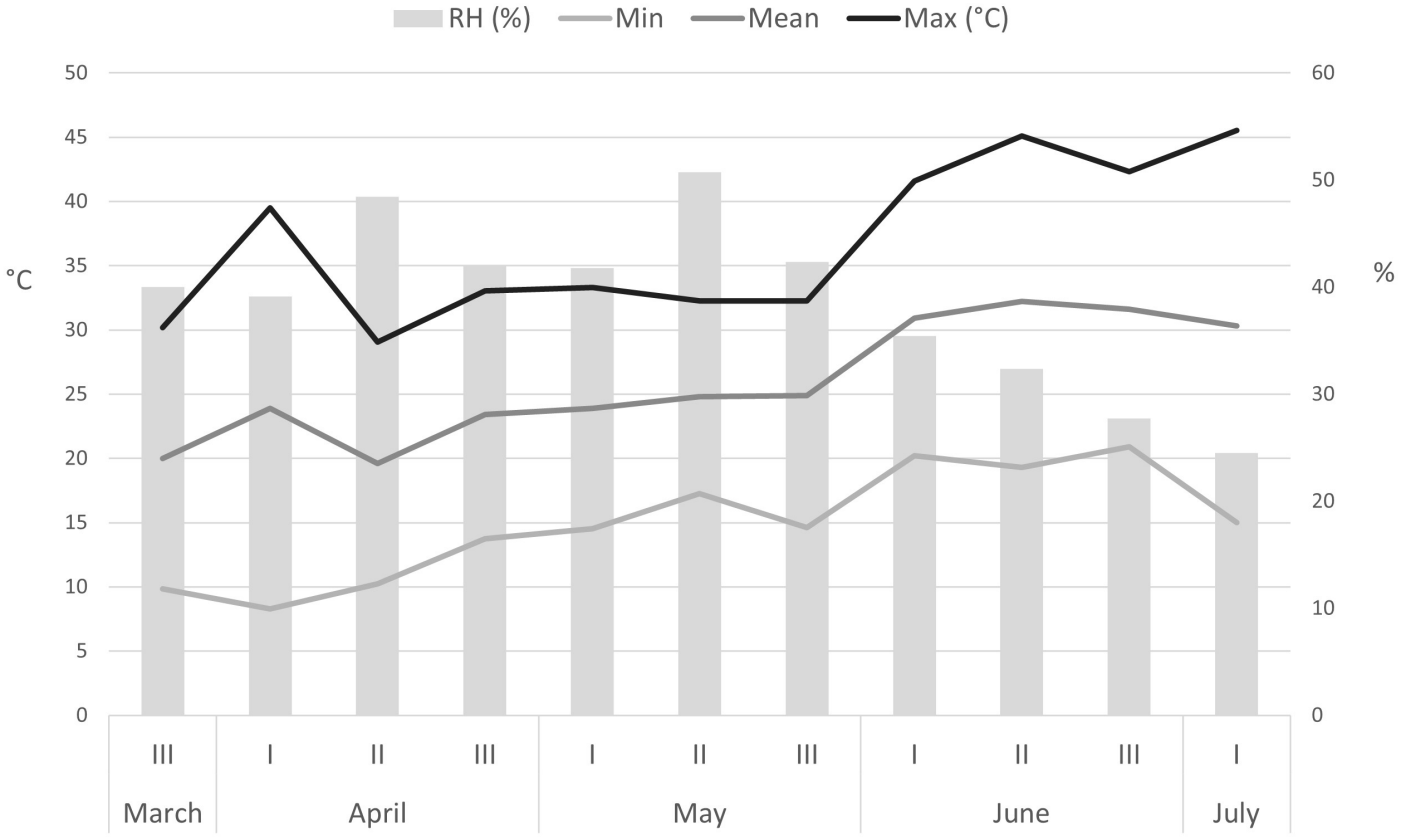
Abiotic factors (temperature and relative humidity) in the greenhouse during hydroponic cultivation of nettle (March 18 – July 5). RH – relative humidity.

**Figure 1** shows greenhouse temperature and relative humidity during stinging nettle cultivation from March 18 to July 5, 2021. Abiotic parameters were measured daily but are shown as mean decadal values.

The minimum air temperature showed a wide range, from 8.3°C in the 1^st^ ten days of April to 20.9°C in the 2^nd^ decade of June. The maximum air temperature, on the other hand, varied from 29.1°C in the 2^nd^ decade of April to a high 45.5°C in the 1^st^ ten days of July. Average air temperature varied from 19.6°C (2^nd^ decade of April) to 32.2°C (2^nd^ decade of June), while relative humidity averaged between 25% and 51%.

The pH and electrical conductivity (EC) of the nutrient solutions are listed in **Table 2**- and were adjusted as required. To balance the pH of the solutions, 56% HNO_3_ was used and it was kept in a range slightly above that recommended for leafy vegetables (5.8 – 6.2), since there are no guidelines for hydroponic cultivation of nettle. In the basin with solution with the weakest nutrient composition (NS1), the average EC value was 1.5 mS/cm and pH varied between 6.6 and 7.2. The EC value in the basin with the nutrient solution of higher nutrient concentration (NS2) was recorded at 1.7 mS/cm, and the pH levels ranged between 6.1 and 7.2. The values in the third nutrient solution (NS3) were 2.9 mS/cm (EC) and 6.5 - 7.3 (pH). In the basin filled with the nutrient solution with the highest concentration of elements (NS4), the average EC value was the highest at 3.0 mS/cm, as it contained the largest quantity of nutrients. The pH of the solution ranged between 6.3 and 7.1.

**Table 2:** Abiotic parameters of nutrient solutions during hydroponic cultivation of nettle shown by decade (April 13 – July 5).

Decades	pH				EC (mS/cm)				DO (mg/L)				T (°C)			
	NS1	NS2	NS3	NS4	NS1	NS2	NS3	NS4	NS1	NS2	NS3	NS4	NS1	NS2	NS3	NS4
April II	7.4	7.2	7.3	7.1	1.9	1.8	2.9	3.0	3.5	3.5	2.0	2.8	17.4	17.4	17.2	17.2
April III	6.8	6.6	6.4	6.4	1.8	1.8	2.8	3.0	2.2	2.0	3.1	2.7	17.8	17.9	18.0	18.0
May I	7.5	7.0	6.7	6.7	1.9	1.8	2.9	3.0	3.4	3.5	3.2	3.5	19.9	19.5	19.6	19.8
May II	7.2	6.8	6.5	6.5	1.4	1.7	2.7	2.9	2.8	2.7	2.0	2.7	23.5	23.2	23.3	23.1
May III	7.2	6.7	6.5	6.5	1.4	1.6	2.8	3.0	3.2	3.0	2.3	2.5	23.6	23.3	23.4	23.3
June I	7.4	7	6.7	6.8	1.2	1.6	2.9	3.1	2.9	2.8	2.4	2.8	27.1	26.9	27.1	27.0
June II	7.2	6.9	6.5	6.6	1.3	1.6	2.8	2.9	3.5	3.6	2.4	3.5	27.5	27.0	27.2	27.4
June III	7.3	6.3	6.6	6.4	1.3	1.7	3.0	3.2	3.4	3.1	3.1	3.5	31.6	31.4	31.9	31.4
July I	7.2	6.1	6.5	6.3	1.3	1.8	2.9	3.1	2.9	2.8	2.6	2.8	31.3	30.8	31.3	30.7

EC, electrical conductivity; DO, diluted oxygen; T, temperature.

**Table 3:** Equations of the calibration curves of the individual phenolic standards.

Standard	Calibration Curve Equation	R^2^
Caffeic acid	y = 13159.9x + 12112.7	0.9998311
Caffeoylmalic acid	y = 20636.8x + 16807	0.999728
Chlorogenic acid	y = 14511.7x – 66325.3	0.9997641
Coumaric acid	y = 2551.11x + 2349.01	0.9998387
Ellagic acid	y = 33829.1x – 6862.97	1.0
Ferulic acid	y = 27461.5 – 90059.3	0.9870226
Kaempferol	y = 88470.8x – 1047410	0.9998243
Naringin	y = 3941.28 – 30329.7	0.9829743
Quercetin 3-glucoside	y = 50449x – 342648	0.9997252
Rutin trihydrate	y = 34107.6x – 238617	0.9989752

During the hydroponic cultivation, the temperature of the solution and the dissolved oxygen (DO) content were also monitored (**Table 2**). The temperature of the solution was lowest in the 1^st^ decade of April (17.2 – 17.4°C) and highest in the 3^rd^ decade of June (31.4 – 31.9°C). The dissolved oxygen levels also varied, ranging from 2.2 - 3.5 mg/L in NS1, 2.0 – 3.6 mg/L in NS2, 2.0 – 3.3 mg/L in NS3, and 2.7 - 3.5 mg/L in NS4, but the solution was considered sufficiently oxygenated because the dissolved oxygen content did not fall below 2.0 mg/L.

The 1^st^ cut occurred on May 20, 2021 (63 days after nettle germination), but due to technical problems resulting in salt deposition in the basins, the plant material from the 1^st^ cut could not be considered relevant to the subject of this study. Due to technical challenges encountered in the initial phase of the experiment within the nutrient solution preparation system, an unintended reaction took place between calcium ions and phosphate and sulfate ions, resulting in sedimentation at the bottom of the basin. These issues were promptly identified and successfully addressed in a timely manner. The consecutive cuts considered in this study were conducted 18 days later, on June 8 (cut I), and 45 days later, on July 5, 2021 (cut II). Manual cutting was done with scissors before the plants reached the flowering stage, and it was done above the lower two nodules to facilitate re-growth.

### 2.2 Number of leaves and yield of hydroponic cultivated nettle

Morphological analyzes were carried out immediately after each cut. The number of leaves was determined by randomly selecting 15 plants per plate, for a total of 30 per replicate or 90 per basins. The total yield was determined by weighing the nettle plant material per plate and expressing it in kg/m². Following yield determination, samples of representative plant material were meticulously prepared, with leaves carefully separated for subsequent analysis of mineral composition and chemical content.

### 2.3 Determination of dry matter, mineral composition and nitrate concentration

Representative samples of the plant material after both cuts, consisting only of leaves without stems, were immediately sent to the Department of the Department of Plant Nutrition. These samples were subjected to thorough analysis for dry matter content, mineral composition, and nitrate concentration.

Nitrates were determined spectrophotometrically after hot water extraction of the fresh samples (AOAC, 2015) and expressed in mg/kg in fresh weight (fw).

For the determination of dry matter content and mineral composition, fresh leaf samples were dried at 105°C to constant weight, then crushed and homogenized. Dry matter was determined by the gravimetric method according to HRN ISO 11465:2004. Total nitrogen in the dry sample was determined by the modified Kjeldahl method (HRN ISO 11261:2004). After digestion of the dry samples with HNO_3_ and HClO_4_, phosphorus was determined spectrophotometrically, potassium by flame photometer, and calcium, magnesium, iron, zinc, maganese, and copper by atomic absorption spectrometer (AOAC, 2015). The dry matter content is expressed in %, the macroelements in % of the dry weight (dw), and the microelements are quantified in mg/kg dw.

### 2.4 Determination of the ascorbic acid content and phenolic compounds

The chemical analyses of ascorbic acid content, phenolic and pigment compounds, and antioxidant capacity, were conducted in the laboratory of the Department of Sustainable Technologies and Renewable Energy Sources. The resultant content values were expressed in mg/g of fresh weight (fw).

The ascorbic acid (AsA) content was determined in accordance with the AOAC (2002) standard method by employing titration with 2,6-dichloroindophenol (DCPIP). Detailed method is described in Dujmović et al., 2023.

For the determination of total phenolics in the samples, the colorimetric reaction with Folin–Ciocâlteu reagent was performed according to Dujmović et al. (2022). The absorbances of the ethanolic extracts were recorded at 750 nm (Shimadzu, 1900i spectrophotometer, Kyoto, Japan). TPC and TNFC results were calculated based on the calibration curve for gallic acid and expressed as milligrams equivalents of the standard per 100 grams of plant fresh weight, while TFC was expressed as the difference between TPC and TNFC and presented as milligrams of catechol equivalents per 100 g of fresh weight.

Phenolic compounds of nettle methanolic extracts were separated using high-performance liquid chromatography (HPLC) Shimadzu system (Shimadzu, Kyoto, Japan), which consisted of a DGU-405 degasser, a LC-40BXR pump, a SIL-40C autosampler, a CTO-40AC column oven, a SPD-M40A photodiode array detector and a RF-20AXS fluorescence detector and a SCL-40 controller. Extract preparation and analysis conditions of separation, identification, and quantification of compounds were the same as in Dujmović et al. (2023). Detection was carried out by scanning over a wavelength range from 220 to 360 nm. The contents of 10 individual phenolic compounds (caffeic, caffeoylmalic, chlorogenic, coumaric, ellagic and ferulic acid, kaempferol, naringin, quercetin 3-glucoside and rutin trihydrate) were expressed on the basis of the calibration curves of the corresponding standards (**Table 3**).

### 2.5 Determination of pigment compounds

Pigment compounds were quantified in accordance with the methods described by Holm (1954) and Wettstein (1957) as shown in Dujmović et al. (2023) and all measurements were performed on a Shimadzu 1900i spectrophotometer (Kyoto, Japan).

### 2.6 Determination of antioxidant capacity

Free radical scavenging activity (ABTS and FRAP) of the ethanolic extracts was measured as described in Dujmović et al. (2023) and all measurements were performed on a Shimadzu 1900i spectrophotometer (Kyoto, Japan). Results were expressed as µmol Trolox equivalents of plant fresh weight.

### 2.7 Statistical analysis

The experiment was set up according to the randomized complete block design with three replicates, where one repetition was represented by two plates of nettle plants randomly placed in basins. In addition, all chemical laboratory analyzes of representative homogenized nettle leaves samples were performed in triplicate. The data obtained were averaged and expressed as mean standard deviation (SD) as shown in the tables. Statistical analyzes of the data were performed using PROC GLM (general linear model) in SAS software system, v. 14.3. (SAS/STAT, 2017), subjected to two-way analysis of variance (ANOVA) and expressed as means, which were compared using a t-test (LSD) and considered statistically significant at a p ≤0.01. Different letters indicate significant statistical differences between samples.

## 3 Results and discussion

### 3.1 Number of leaves and yield of nettle

The results of the number of leaves and yield of the studied nettle plants grown in four nutrient solutions of different composition during two consecutive cuts are shown in **Table 4**. Statistical analysis showed the nutrient solution (NS) and the interaction of the two factors (NS × C) significantly influenced the number of leaves per plant. However, the influence of cut (C) had no statistically justified effect on this morphological trait. Yield was significantly influenced by both factors and their interactions.

**Table 4:** Number of leaves and yield of nettle cultivated in different nutrient solutions.

Treatment	Number of leaves	Yield (kg/m^2^)
NS1 × C I	11.00^abc^ ± 1.40	2.30^ab^ ± 0.27
NS2 × C I	11.40 ^abc^± 0.12	1.82^b^ ± 0.32
NS3 × C I	10.87^abc^ ± 1.71	2.49^a^ ± 0.36
NS4 × C I	8.47^c^ ± 1.72	1.99^ab^ ± 0.30
NS1 × C II	12.00^ab^ ± 1.70	0.44^c^ ± 0.27
NS2 × C II	12.27^a^ ± 1.30	0.53^c^ ± 0.05
NS3 × C II	8.90^bc^ ± 1.11	0.58^c^ ± 0.17
NS4 × C II	11.47^abc^ ± 0.96	0.41^c^ ± 0.01
ANOVA	0.0292	p ≤ 0.0001
NS	0.0343^*^	0.0976^ns^
C	0.1513^ns^	0.0001^***^
NS × C	0.0292^*^	0.0001^***^

NS, nutrient solution; C, consecutive cut; NS × C - the interaction of nutrient solution and consecutive cut; C I, 1^st^ cut; C II, 2^nd^ cut. Results are expressed as mean ± standard deviation. Different letters indicate significant differences between mean values. ns, * and *** indicate significant effects at p < 0.05 and 0.001, respectively.

The number of leaves is an important morphological trait because it has a substantial impact on the total plant yield. In this study, the number of leaves varied from 8.47 to 12.27 per plant. The average number of leaves was 10.43 after the cut I (C I) and 11.16 after the cut II (C II). Radman et al. (2014) determined a comparable number of nettle leaves and noted a trend toward an increase in leaf number during the 2^nd^ consecutive cut when nettle was grown in a floating system. However, when grown in an open field, a greater number of stinging nettle leaves were observed than in the floating hydroponics (Radman et al., 2022). In this research, after the C I the lowest number of leaves was observed in NS4 (8.47), although it was a solution with the strongest nutrient composition, especially in terms of the content of nitrogen and potassium, elements important for plant growth. This weaker uptake of biogenic elements by the nettle, which grew in a stronger solution, could be explained by the presence of salt deposits in the basins under the influence of high temperatures; the temperature of the nutrient solution varied between 17.2 and 31.4°C, as evident in **Table 2**. The nettle from NS2 had the highest number of leaves after both consecutive cuts (11.40 and 12.27). In contrast to the number of leaves, in the same NS a higher average yield was observed after the C I (2.49 kg/m^2^), even four times more than after the C II (0.41 kg/m^2^).

### 3.2 Dry matter content of nettle

Nutrient solution, consecutive cuts, and interactions (**Table 5**) had a statistically significant influence on a nettle dry matter (DM) content. Justifiably the highest content of DM was determined in the plants from treatment NS2 × C II while lowest DM content was found in nettle from C I in treatments NS4 and NS3 (16.05% and 14.56%). A statistically equal share of DM was achieved in the C II in the treatments NS1, NS3 and NS4 (24.34%, 24.16% and 24.24%). Treatments NS2 and NS4 in C I were also statistically in the same rank. In general, higher DM values were found in plant material from the C II which is consistent with fact that DM increases with plant ageing.

**Table 5:** Dry matter content, mineral composition and nitrate concentration of nettle cultivated in different nutrient solutions.

Treatment	DM	N	P	K	Ca	Mg	Fe	Zn	Mn	Cu	NO_3_^-^
		%					mg/kg				
NS1 × C I	16.81^c^ ± 0.35	6.32^b^ ± 0.03	0.61^b^ ± 0.01	3.89^c^ ± 0.02	5.54^a^ ± 0.08	1.03^c^ ± 0.06	100.00^f^ ± 3.61	34.43^b^ ± 0.76	35.43^d^ ± 0.87	25.00^b^ ± 0.56	6196.67^b^ ± 287.29
NS2 × C I	16.58^cd^ ± 0.33	6.21^b^ ± 0.03	0.66^a^ ± 0.02	3.78^c^ ± 0.09	4.24^c^ ± 0.02	1.46^a^ ± 0.01	121.00^e^ ± 1.00	24.20^e^ ± 0.36	80.70^b^ ± 2.51	29.73^a^ ± 0.35	5366.67^c^ ± 52.84
NS3 × C I	14.56^e^ ± 0.18	6.70^a^ ± 0.09	0.70^a^ ± 0.03	4.64^a^ ± 0.16	4.69^b^ ± 0.08	0.81^e^ ± 0.03	136.67^d^ ± 3.06	36.03^a^ ± 0.32	140.80^a^ ± 3.48	21.93^c^ ± 0.86	6117.00^b^ ± 202.90
NS4 × C I	16.05^d^ ± 0.20	6.20^b^ ± 0.05	0.59^b^ ± 0.03	4.38^b^ ± 0.09	4.42^c^ ± 0.13	0.88^de^ ± 0.07	115.67^e^ ± 5.13	27.50^c^ ± 1.11	145.53^a^ ± 2.93	10.90^d^ ± 0.20	6202.67^b^ ± 70.19
NS1 × C II	24.34^b^ ± 0.03	3.97^e^ ± 0.06	0.26^d^ ± 0.01	3.07^e^ ± 0.03	3.17^d^ ± 0.22	0.83^de^ ± 0.02	180.67^a^ ± 5.69	23.13^e^ ± 0.25	24.10^f^ ± 1.05	4.63^f^ ± 0.85	4225.33^d^ ± 215.98
NS2 × C II	25.25^a^ ± 0.16	3.96^e^ ± 0.03	0.36^c^ ± 0.01	3.33^d^ ± 0.07	2.26^f^ ± 0.09	1.23^b^ ± 0.03	160.33^c^ ± 2.89	12.43^g^ ± 0.80	32.03^de^ ± 2.60	8.60^e^ ± 0.36	4946.67^c^ ± 314.10
NS3 × C II	24.16^b^ ± 0.41	4.96^c^ ± 0.04	0.35^c^ ± 0.01	4.53^ab^ ± 0.06	2.82^e^ ± 0.09	0.89^de^ ± 0.01	170.67^ab^ ± 7.37	25.77^d^ ± 0.74	50.13^c^ ± 3.23	11.10^d^ ± 0.36	7153.33^a^ ± 85.36
NS4 × C II	24.24^b^ ± 0.13	4.77^d^ ± 0.02	0.37^c^ ± 0.01	4.36^b^ ± 0.06	2.88^e^ ± 0.08	0.92^d^ ± 0.02	162.00^bc^ ± 4.58	17.40^f^ ± 0.10	27.63^ef^ ± 1.10	4.60^f^ ± 0.30	6050.00^b^ ± 114.35
ANOVA	p ≤ 0.0001	p ≤ 0.0001	p ≤ 0.0001	p ≤ 0.0001	p ≤ 0.0001	p ≤ 0.0001	p ≤ 0.0001	p ≤ 0.0001	p ≤ 0.0001	p ≤ 0.0001	p ≤ 0.0001
NS	0.0001^***^	0.0001^***^	0.0001^***^	0.0001^***^	0.0001^***^	0.0001^***^	0.0002^***^	0.0001^***^	0.0001^***^	0.0001^***^	0.0001^***^
C	0.0001^***^	0.0001^***^	0.0001^***^	0.0001^***^	0.0001^***^	0.0001^***^	0.0001^***^	0.0001^***^	0.0001^***^	0.0001^***^	0.0003^***^
NS × C	0.0001^***^	0.0001^***^	0.0001^***^	0.0001^***^	0.0001^***^	0.0001^***^	0.0001^***^	0.0001^***^	0.0001^***^	0.0001^***^	0.0001^***^

DM, dry matter; NO_3_^-^ - nitrate content; NS - nutrient solution; C, consecutive cut; NS × C - the interaction of nutrient solution and consecutive cut; C I, 1^st^ cut; C II, 2^nd^ cut. Results are expressed as mean ± standard deviation. Different letters indicate significant differences between mean values.*** indicate significant effects at p < 0.001.

Dry matter is an important indicator of plant material quality because, it contains a variety of vital components, including proteins, carbohydrates, vitamins, minerals, specialized metabolites, and other substances besides water. Therefore, plant material with higher DM values reflects better nutritional quality. In a study by Biesiada et al. (2010), which examined the effects of plant age and harvest period on the chemical composition and antioxidant activity of nettle (cultivated in open field), DM values averaged 28.34%, slightly higher than hydroponic nettle in this study. Rutto et al. (2013) report 11 – 24.9% of DM in fresh nettle leaves cultivated in open field in spring and fall period. The dry matter of nettle leaves in the 6^th^ harvest from the study by Radman et al. (2015) averaged 23.82%, which was similar to our results of the C II.

### 3.3 Mineral composition of nettle

According to various studies (Rutto et al., 2013; Upton, 2013; Đurović et al., 2017; Kregiel et al., 2018), the nettle plant is an excellent source of minerals, so it can be recommended as part of a diverse and balanced diet rich in vitamins, minerals and other health-promoting substances. Therefore, the application of modern technologies plays an important role in the cultivation of plant species with the aim of increasing the nutritional value of the final product. However, during cultivation, factors such as pH, redox potential, biological activity, and cation exchange capacity can affect the uptake of nutrients, especially microelements. In addition, plant factors such as root and root hair morphology, root-induced changes, exudation of organic acids, sugars, and non-proteinogenic amino acids, secretion of enzymes, plant requirements, and plant species/cultivars have a profound effect on the ability of plants to take up and utilize nutrients (Fageria et al., 2002). Minerals are often divided into macro- and microelements, depending on the amounts required for the normal functioning of the organism.

**Table 5** shows the result of dry matter, macro and microelements and nitrate concentration of nettle plants cultivated in different NSs.

Nitrogen (N) is a main component of several essential compounds, including amino acids, nucleic acids and proteins which are vital for various physiological functions in the human body, including tissue growth, immune system function, enzyme activity, and the maintenance and repair of cells (Bian et al., 2020; Nieder and Benbi, 2022). The treatment that resulted in higher N concentration in nettle was NS3 × C I [6.70% N in dry weight (dw)], while the lowest N concentration in nettle was achieved in C II by treatments NS1 and NS2 (3.97 and 3.96% N dw). This was to be expected considering the lowest concentration of N in NS1 and NS2. Treatments NS1, NS2 and NS4 in C I yielded statistically the same N content. Solutions NS3 and NS4 had twice as much nitrogen in their formulation, but this trend was not evident in the plant material. In the study by Radman et al. (2021) on the mineral content of hydroponically grown nettle, the nitrogen content was 3.87–4.17% N dw, which are somewhat lower values than in this study, especially the values found in the C I. Biesiada et al. (2010) also reported lower N content (3.30 – 3.67% N dw) in nettle grown in the open field.

Phosphorus (P) is essential for a variety of functions, including energy metabolism (as a component of ATP), enzyme activity, nucleic acid synthesis, and the assembly of cell membranes (phospholipids), phosphoproteins, and coenzymes. It is also involved in the metabolism of red blood cells. Although phosphorus is essential, it is noted that the intake of phosphorus by the general population often exceeds the amount required for health due to phosphate-containing additives in processed foods (Bird and Eskin, 2021). Highest values of P were detected in nettle from C I in treatments NS2 and NS3 (0.66 and 0.70% P dw) and treatment NS1 × C II resulted in almost 2.7 times lower content of P while treatments NS2, NS3 and NS4 in C II didn’t statistically differ (0.36, 0.35 and 0.37% P dw). If we look at the composition of the NSs, it would be expected for the plants grown in NS2 and NS4 to have a higher phosphorus content. A slightly higher P content was determined by Paulauskienė et al. (2021) in stinging nettles grown in the field, reporting 0.81% P dw. In contrast, Biesiada et al. (2010) reported 0.37-0.40% P dw, which is similar to the values found in the C II of this study.

Potassium (K) is necessary for muscle cell function, and a lack of it can lead to muscle cramps, weakened bowel function, and heart problems. It also serves as a cofactor for enzymes such as pyruvate kinase. Potassium plays an important role in fluid balance, muscle contraction, transmission of nerve impulses and regulation of blood pressure but many people consume less potassium than recommended (McLean and Wang, 2021). Same as in the case of N content, nettle plants from treatment NS3 in C I had the highest values of K ranging 4.64% K dw while NS1 × C II resulted in plant material with lowest K content (3.07% K dw). Value of K in treatment NS3 × C II (4.53% K dw) did not differ from values achieved by treatments NS4 in C I (4.38% K dw) and NS4 in C II (4.36% K dw). Solution NS3 was the one with the highest K content in the formulation, so the highest K content would be expected in treatments NS3 × C I and NS3 × C II. According to Đurović et al. (2017), the K content in wild-harvested stinging nettle is 33899.00 μg/g K. Radman et al. (2021) found 4.30 - 4.45% K dw in nettle from floating hydroponics, which is consistent with the results of this study, while nettle from open field cultivation contained 50% less K in the study by Radman et al. (2015). Lower content of K in stinging nettles from field cultivation were also reported in the study by Biesiada et al. (2010).

Calcium (Ca) is one of the most abundant elements in the human body and is best known for its role in building bones and teeth. However, it has a variety of functions, including muscle contraction, nerve transmission, blood clotting, regulation of heart rhythm, and maintenance of fluid balance in cells. Ca absorption can be affected by factors such as vitamin D, manganese and fluoride. Inadequate calcium intake can result from excessive consumption of acidic and canned foods and NaCl (Pravina et al., 2013). Nettle from treatment NS1 × C I had highest Ca content (5.54% Ca dw) while treatment NS2 in C I resulted in nettle with almost 60% less Ca although NS1 and NS2 formulation had similar content of Ca(NO_3_)_2_. In general, nettle plants in C II had lower content of Ca in comparison to C I. This was not to be expected, as these higher values were determined in a solution with a weaker composition and in younger plants. This phenomenon can possibly be explained by the presence of stress conditions (abiotic factors). As a result of the high temperatures (**Figure 1**, **Table 2**), there was a potential salt deposition in solutions with a higher concentration, so that the plants could not take up calcium. The Ca content of wild harvested nettle from Macedonia ranged from 2.63 to 5.09% Ca dm, which is consistent with the results of this study. The results of the studies by Biesiada et al. (2010); Paulauskienė et al. (2021), and Radman et al. (2021) are also in this range, while Đurović et al. (2017) report a value of 28.605 μg/g Ca.

Magnesium (Mg) is a cofactor for over 300 enzymes involved in various biochemical reactions in the body. It plays a critical role in processes such as protein synthesis, muscle and nerve function, blood sugar control, and blood pressure regulation. Mg is needed for energy production, bone formation, DNA and RNA synthesis, and antioxidant activity (e.g., glutathione). A Mg deficiency can lead to problems such as muscle and heart problems, digestive disorders, concentration problems, irritability, fatigue, depression and increased adrenaline (Glasdam et al., 2016). Treatment NS2 in C I resulted in plants with highest values of Mg with content of 1.46% Mg dw. In the same cut plants from treatment NS3 had lowest Mg content (0.81% Mg dw) although NS3 formulation had the same content of MgSO_4_. When we look at the C II only, it is evident that same treatment (NS2) produced plants with higher Mg content in comparison to the other three solutions. The average Mg content in this study (data not shown) was 50% higher than that reported by Radman et al. (2021) for nettle cultivated in floating hydroponics. On the other hand, Rafajlovska et al. (2013) found 2.51-3.56% Mg dw in wild harvested nettle plant material. The content found in nettle from the study by Đurović et al. (2017) was 8699.76 μg/g Mg.

The results of the content of microelements of the studied nettle plants grown during two consecutive cuts under different nutrient treatments are shown in **Table 5**. Statistical analysis showed that both tested factors (NS and C) and their interaction (NS × C) significantly (≤ 0.0001) affected the content of microelements (iron, zinc, manganese, copper) of nettle plants.

Micronutrients are needed by the body in very small amounts. However, their impact on the health of the body is crucial, and a deficiency of any of them can lead to serious and even life-threatening diseases. Many of these deficiencies can be prevented by a healthy, varied diet (WHO, 2023). Iron deficiency anemia is a global nutritional concern that predominantly affects children and pregnant women, as highlighted by the World Health Organization (WHO) in 2023. Therefore, it is imperative to conduct research aimed at promoting the consumption of iron-rich foods. Previous studies (Upton, 2013; Adhikari et al., 2016; Radman et al., 2016; Kregiel et al., 2018; Paulauskienė et al., 2021; Radman et al., 2021) have unequivocally demonstrated the iron-rich nature of nettle. The iron content varied depending on factors such as the nettle’s origin (wild or cultivated), cultivation methods (e.g., soil or hydroponics), and geographical location. In the present study, the iron (Fe) content ranged from 100.00 to 180.67 mg/kg of dry matter, reaffirming nettle’s classification as an iron-rich plant species. On average, higher Fe levels were observed during the C II (168.42 mg/kg Fe dw) compared to the C I (118.34 mg/kg Fe dw); data not shown. The influence of nutrient solutions, however, varied depending on the consecutive cut. During the C I, nettles cultivated in NS3 exhibited the highest Fe content (136.67 mg/kg Fe dw), followed by NS2 and NS4, resulting in decreased Fe content in nettle leaves (121.00 and 115.67 mg/kg Fe dw, respectively). Nettles grown in NS1 had the lowest Fe content (100.00 mg/kg Fe dw), which aligns with the fact that NS1 contains the lowest iron concentration (12.8 mg/L FeEDTA 13%). Consequently, the plants absorbed the least iron from this solution. In contrast, NS4 contains nearly four times the Fe concentration (42.68 mg/L FeEDTA 13%). Surprisingly, the plants did not absorb a larger quantity, potentially due to NS4 higher nitrogen content, which could have affected the nettle’s Fe uptake. This aligns with the findings of Radman et al. (2016), who reported a negative impact of nitrogen fertilization on Fe uptake by plants. In the C II, the iron levels in the tested nutrient solutions ranked as follows: NS2 (160.33 mg/kg Fe dw) > NS4 (162.00 mg/kg Fe dw) > NS3 (170.67 mg/kg Fe dw) > NS1 (180.67 mg/kg Fe dw). According to Brown (1978), the pH of the nutrient solution also has an impact on the Fe uptake. This means that even if iron is present in the soil or nutrient solution, it may not be readily available to plants if the pH is not within the appropriate range (alkaline environment).

The zinc (Zn) content in the nettle varied from 12.43 to 36.03 mg/kg Zn dw, with the plants containing more Zn on average after the C I (30.54 mg/kg Zn dw) than after the C II (19.68 mg/kg Zn dw); data not shown. As for the NS, after both consecutive cuts, nettles contained more Zn when grown in the NS3 (36.03 and 25.77 mg/kg Zn dw), while the NS2 proved to be the least favorable (24.20 and 12.43 mg/kg Zn dw). Clearly, a decline in Zn content is observed across all four nutrient solutions during the C II. This reduction is particularly conspicuous in the case of the NS2, where the Zn levels in the C II are only about half of their previous values. This drop can be ascribed to the greenhouse and nutrient solution’s rising temperatures, as clearly depicted in **Table 2** and **Figure 1**. The elevated temperatures within the nutrient solution directly impact root metabolism, leading to diminished respiration and ATP production. ATP, being essential for the active absorption of nutrients by plant roots, plays a critical role in this process (Bergmann, 1992; Lazarević and Poljak, 2019).

The manganese (Mn) content in nettles exhibited a range from 24.10 mg/kg Mn dw to 145.53 mg/kg Mn dw. On average, following the C I, the plant material contained Mn at an average of 100.62 mg/kg Mn dw, but this dropped to just 33.47 mg/kg Mn dw after the C II (data not shown). This trend closely resembles that of iron but contrasts with the pattern observed for zinc. Mn is primarily absorbed through diffusion, as outlined by Bergmann (1992). Consequently, the increased temperatures and heightened humidity observed during C II led to decreased transpiration, ultimately resulting in less efficient Mn uptake and reduced involvement in metabolic processes. In the C I, the highest Mn values were achieved by applying NS4 (145.53 mg/kg Mn dw) and NS3 (140.80 mg/kg Mn dw). This was to be expected, since these two solutions contain the highest content of manganese in their composition compared to the other two solutions (**Table 1**). Subsequently, Mn content declined in the remaining two solutions, with NS2 measuring at 80.70 mg/kg Mn dw and NS1 at 35.43 mg/kg Mn dw. NS3 was most favorable for stinging nettle in the C II (50.13 mg/kg Mn dw), while stinging nettle in NS2 (32.03 mg/kg Mn dw), NS4 (27.63 mg/kg Mn dw), and NS1 (24.10 mg/kg Mn dw) contained the least Zn after the C II.

Copper (Cu) levels in the samples exhibited a range from 4.60 mg/kg Cu dw (in NS4 × C II) to 29.73 mg/kg Cu dw (in NS2 × C I). On average, Cu levels were three times higher after the C I (21.89 mg/kg Cu dw) compared to the C II (7.23 mg/kg Cu dw); data not shown. Examining the impact of nutrient solutions on Cu levels in nettle, we find that during the C I, there was an increase in Cu content as follows: NS4 (10.90 mg/kg Cu dw) > NS3 (21.93 mg/kg Cu dw) > NS1 (25.00 mg/kg Cu dw) > NS2 (29.73 mg/kg Cu dw). This trend persisted during the C II, with the lowest Cu content recorded in NS4 (4.60 mg/kg Cu dw) and statistically the same value in NS1 (4.63 mg/kg Cu dw). Cu levels in nettle leaves increased when using NS2 (8.60 mg/kg Cu dw) and NS3 (11.10 mg/kg Cu dw). This outcome was anticipated since these two solutions are richer in this microelement, while NS1 contains the least copper.

### 3.4 Nitrate concentration of nettle

**Table 5** also shows the results of nitrate concentration in nettle leaves expressed in fresh weight (fw), and significant differences were found among the values. The highest nitrate concentration was found in nettle from C II in treatment NS3 (7153.33 mg NO_3_/kg fw), while treatment NS1 gave the lowest nitrate value, almost 40% less (4225.33 mg NO_3_/kg fw). Treatments NS1, NS3 and NS4 in C I and NS4 in C II were not statistically different from each other regardless of the different content of nitrogen available in NSs.

Leafy vegetables tend to accumulate nitrates, so reducing nitrate levels is an important aspect of vegetable production (Gonnella et al., 2004; Nicola et al., 2006; Luetic et al., 2023). Currently, there is no legal framework in the EU for the maximum content of nitrates allowed in fresh nettle leaves, but there is a regulation for green leafy vegetables such as spinach – *Spinacia oleracea* L. (which consumers are known to substitute for nettle when preparing meals) and arugula – *Eruca sativa* L. According to Commission Regulation 2023/915 of April 25, 2023 (European Commission, 2023b) on maximum levels for certain contaminants in food, the maximum level of nitrates in fresh spinach is 3500 mg NO_3_/kg fw. For arugula, the maximum levels range from 6000 to 7000 mg NO_3_/kg fw, depending on the growing period. The results obtained in this study for nettle are below or within the legal limits of the values for arugula. The uptake and accumulation of nitrates in vegetables is influenced by numerous factors such as humidity, temperature, radiation, photoperiodism, amount and formulation of nitrogen fertilizer, and availability of other nutrients such as phosphorus, potassium, calcium, iron, or molybdenum. High temperatures are the biggest influencing factor, leading to increased transpiration and consequently higher nitrate levels in the plant. During the nettle cultivation in floating hydroponics, the temperature of the NS was the limiting factor, as considerable differences were observed given the considerable variation from May to July. In the C I (May 21-June 7), the temperature of NS was between 23.5 and 27°C, and during C II (June 8–July 4), the temperature of NS increased to 30 and 32°C. The optimal temperature range for nettle growth is between 20 and 25°C (Opačić et al., 2022), so high temperatures certainly resulted in higher nitrate levels in nettle. The lowest nitrate level detected in nettle cultivated in NS1 during the C II might be due to the fact that NS1 had lower nitrogen concentration than NS3 and NS4, which contained up to 2 times more nitrogen. Some research suggests (Gonnella et al., 2004; Nicola et al., 2006; Radman et al., 2016) that nitrate levels can be lowered by replacing the NS with water a few days before harvest. In addition, nitrate content can be reduced by blanching (Wu et al., 2021). Since nettle leaves are heat treated before consumption (because they are covered with stinging hairs), this could be the way to further reduce nitrate levels in this type of plant material. Nitrate levels in stinging nettles grown in open field and treated with varying amounts of nitrogen fertilizer ranged from 780 to 2070 mg NO_3_/kg fw in the study by Radman et al. (2015). These values are much lower than those found in this study, while Biesiada et al. (2010) reported even lower values.

### 3.5 Ascorbic acid content of nettle

According to the results of ascorbic acid content (AsA) in fresh nettle leaves (**Table 6**), the highest AsA content was observed after C II in treatment with NS2 with the value of even 102.3 mg/100 g fw. The AsA content in nettle leaves was strongly influenced by the nutrient solution content, as shown by the results of the significance of each varied factor for NS p ≤ 0.0001 and the combination of nutrient solution content and consecutive cuts (NS × C p ≤ 0.0004). In general, regardless of the content of the nutrient solution, higher average AsA content was determined after C II compared to the C I. Those differences were not so pronounced, but statistically justified with about 5% higher AsA values in C II to C I. Nutrient solution content had a significant impact on AsA values, while in C I highest AsA content was observed in nettle leaves treated by NS4, while no significant differences were determined between NS1 and NS3 with the lowest determined AsA content in leaves from C I. On average, the difference between the highest and lowest AsA values in the C I amounted to 23%. Furthermore, in C II, highest AsA content was determined in treatment with NS2, while the lowest with no significant difference, in treatments with NS3 and NS4 with average value of 73.82 mg/100 g fw (data not shown). The difference between the highest and lowest AsA values in the C I amounted to 39%.

**Table 6:** Specialized metabolites content of nettle cultivated in different nutrient solutions.

Treatment	ASA (mg/100 g)	TPC (mg GAE/100 g)	TNFC (mg GAE/100 g)	TFC (mg CTH/100 g)	Ant_cap FRAP (μmol TE/L)	Ant_cap ABTS (μmol TE/L)
NS1 × C I	74.90^c^ ± 7.07	215.82^e^ ± 1.88	71.48^g^ ± 1.20	144.34^e^ ± 2.88	1224.77^f^ ± 19.12	2423.04^bcd^ ± 9.72
NS2 × C I	80.25^bc^ ± 7.59	238.39^d^ ± 1.43	97.78^e^ ± 0.53	140.61^e^ ± 1.90	1554.40^e^ ± 17.99	2413.83^cd^ ± 21.26
NS3 × C I	69.73^c^ ± 4.00	164.79^g^ ± 3.31	85.88^f^ ± 0.43	78.92^g^ ± 2.89	963.27^h^ ± 11.92	2461.24^bc^ ± 4.35
NS4 × C I	89.03^b^ ± 3.35	170.23^f^ ± 0.91	73.28^g^ ± 2.91	96.94^f^ ± 2.03	1078.05^g^ ± 9.06	2442.40^bc^ ± 9.89
NS1 × C II	79.38^bc^ ± 6.68	419.22^b^ ± 0.63	205.16^c^ ± 2.31	214.06^b^ ± 1.67	3122.07^b^ ± 41.36	2475.76^ab^ ± 5.40
NS2 × C II	102.30^a^ ± 1.19	465.92^a^ ± 0.45	225.16^a^ ± 0.57	240.69^a^ ± 0.95	3237.62^a^ ± 21.20	2525.78^a^ ± 7.68
NS3 × C II	72.78^c^ ± 4.32	352.24^c^ ± 0.89	164.31^d^ ± 1.08	187.93^d^ ± 1.18	2617.92^d^ ± 20.92	2467.46^abc^ ± 17.75
NS4 × C II	74.86^c^ ± 4.67	420.56^b^ ± 1.75	211.78^b^ ± 0.39	208.78^c^ ± 1.91	3033.61^c^ ± 14.76	2365.24^d^ ± 63.78
ANOVA	p ≤ 0.0002	p ≤ 0.0001	p ≤ 0.0001	p ≤ 0.0001	p ≤ 0.0001	p ≤ 0.0002
NS	0.0001^***^	0.0001^***^	0.0001^***^	0.0001^***^	0.0001^***^	0.0013^***^
C	0.1001^ns^	0.0001^***^	0.0001^***^	0.0001^***^	0.0001^***^	0.0346^*^
NS × C	0.0004^***^	0.0001^***^	0.0001^***^	0.0001^***^	0.0001^***^	0.0001^***^

NS, nutrient solution; C, consecutive cut; NS × C, the interaction of nutrient solution and consecutive cut; C I, 1^st^ cut; C II, 2^nd^ cut; AsA, ascorbic acid content; TPC, total phenol content; TNFC, total non-flavonoid content; TFC, total flavonoid content; Ant_cap, antioxidant capacity. Results are expressed as mean ± standard deviation. Different letters indicate significant differences between mean values. * and *** indicate significant effects at p < 0.05 and 0.001, respectively.

The nettle species is characterized by the fact that it can regrow during the cultivation, i.e. had possibility to re-growth. Therefore, during harvest it must be cut above the first two nodules so that the plants can regrow. Depending on various factors, mainly environmental (temperature, humidity) and agrotechnical (cultivation method, irrigation, nutrient availability), nettle can reach several consecutive cuts during the cultivation period without significant changes in morphological characteristics, especially without loss of total yield and pronounced impairment of the dynamics of plant growth, leaf size, etc (Radman, 2015). The most important in determining the time of harvest is to avoid the flowering phase, i.e. the generative phase, in order to allow the regrowth of the plant and also to preserve the content of important secondary plant compounds. One of the compounds that play an important role in plant growth and development is vitamins, which even in very small amounts can have a pronounced effect as bioregulators and precursors of hormones such as cytokines and gibberellins, which are important for the development and growth of the plant organism. More specifically, AsA has several important functions in the plant organism, including reducing cell division, mitigating oxidative stress in plant cells (antioxidant properties), it is a co-factor for enzymes and a precursor for oxalate and tartrate synthesis (Gomes et al., 2014; Naz et al., 2016; Gaafar et al., 2020). In addition, AsA affects energy metabolic pathways by protecting them from the effects of H_2_O_2_ and other oxygen free radicals through its scavenging action or by regenerating the reduced forms of tocopherols or zeaxanthin (Hasanuzzaman et al., 2020; Šic Žlabur et al., 2021). The process of harvest is perceived by plants as a kind of stress, and they begin to activate defense mechanisms in which AsA plays one of the main roles as a powerful antioxidant. The availability of AsA to the plant organism at the moment of stress has significant effects on almost all-important physiological processes in the plant organism, from photosynthesis to the biosynthesis of compounds of secondary metabolism to cell division (Akram et al., 2017). Considering the above, the general tendency of higher AsA content in nettle leaves after C II compared to C I can be explained by the effect of AsA protecting plants from oxidative stress caused by consecutive cuts. Other research studies also demonstrate the effect of multiple harvesting in the growing season on AsA content in plants, finding a positive effect of AsA accumulation in plants that are harvested multiple times during cultivation (Lee and Kader, 2000; Radman et al., 2015). Moreover, the content of the NS significantly affected the AsA content in the nettle leaves despite the number of harvests. Biogenic elements, which are crucial for normal plant growth and development, also significantly affect the accumulation of phytochemicals in various plant organs. As a nitrophilous species (Opačić et al., 2022) nettle shows a significant tendency to accumulate nitrates from the nutrient solution, with nitrogen content showing a pronounced influence on AsA accumulation along with other various factors such as stress, ecological factors, cultivation practices, etc. (Roumeliotis et al., 2021; Chadzinikolau and Formela-Luboińska, 2023). The nutrient solutions in this study mainly varied in nitrogen content, with NS1 containing the lowest nitrogen content, while NS4 the highest nitrogen content. In general, nitrogen content negatively affects AsA accumulation, while excess nitrogen leads to lower synthesis of AsA in plant tissues (Roumeliotis et al., 2021; Chadzinikolau and Formela-Luboińska, 2023). In C I some contrary results were obtained since the highest AsA content was recorded in plants treated by NS4, while lowest in leaves treated by NS1 and NS3. Given stated, some uniformity in the results cannot be distinguished, i.e. it cannot be clearly confirmed that NS with a higher nitrogen content result in a lower AsA content and vice versa. This might be caused by the impact of other biogenic elements from NS, since they show a synergistic effect, where the lack of one can affect the increase of the other and vice versa. For example, AsA content is positively related to the concentration of Mg and P (Abanto-Rodriguez et al., 2016). In C II, opposite results were obtained, with the lowest AsA content in the plants treated with NS4 (highest content of nitrogen) and the highest AsA content in the leaves treated with NS2 (low nitrogen content). Considering other studies that have analyzed the AsA content in nettle leaves, it can be stated that the AsA values obtained in this study are in agreement with other literature data or even higher, depending strongly on the cultivation practice, the origin of the plant and, of course, ecological factors during the cultivation period (Radman et al., 2015; Paulauskienė et al., 2021; Opačić et al., 2022).

### 3.6 Phenolic content of nettle

Phenolic compounds are a diverse group of phytochemicals that play several crucial roles in plants, including protection against various stress conditions (Chowdhary et al., 2021). These effects are manifested thanks to the antioxidant activity of phenols which contributes to plant development overall but also has beneficial biological effects on human organisms (Nardini, 2022).

The results of the total phenolics (TPC) of the studied nettle plants grown under different nutrition treatments during two consecutive cuts are presented in **Table 6**. According to the statistical analysis, both tested factors (NS and C) and their interaction (NS × C) significantly affected (≤ 0.0001) total phenolics content. The TPC values varied from 164.79 to 465.92, and TFC from 78.92 to 240.69 mg GAE/100 g fw. Overall results show that the highest content of total phenolics was observed at treatment NS2 × C II. All nutrient elements present in NSs significantly influence the phenolic composition in plants by impacting the biosynthesis, accumulation, and distribution of phenolic compounds (Heimler et al., 2017; Bustamante et al., 2020; Ebrahimian et al., 2021; Vilkickyte and Raudone, 2021) and nutrient levels and forms in solution can impact the quality and quantity of phenolics as well (Vilkickyte and Raudone, 2021; Marlin et al., 2022). However, nitrogen is a major essential nutrient for plant growth and development, which also significantly influences the phenolic composition in plants. Some studies (Radušienė et al., 2019; Marlin et al., 2022) show that higher nitrogen availability and nitrogen-rich environments in general may suppress the accumulation of certain phenolic compounds. On the other hand, plants exposed to N deficiency often respond by increasing the synthesis and accumulation of phenolic compounds, as a defense mechanism to cope with the stress caused by nutrient deficiency (Nguyen and Niemeyer, 2008; Fortier et al., 2010; Stefanelli et al., 2010; Heimler et al., 2017) which goes in favor with the results obtained in this study. The content of N in tested NSs are as follows: NS4 > NS3 > NS2 > NS1. In this study, regardless of the consecutive cut, the highest values for TPC were recorded while applying NS1. As an essential macronutrient for plant functions, a certain content of N is needed, so the N content in NS1 as obviously too low for higher total phenol accumulation. NS2 has a lower N content than NS3 and NS4, confirming that greater phenol production can occur as a lack of nitrogen, which also coincides with the aforementioned research of Nguyen and Niemeyer (2008); Fortier et al. (2010); Stefanelli et al. (2010); Heimler et al. (2017); Radušienė et al. (2019) and Marlin et al. (2022). Also, in a study by Radman et al. (2015) higher phenolic values of nettle leaves were recorded by applying lower N fertilization and high N levels significantly reduced the concentration of TFC of nettle aerial parts according to Grevsen et al. (2008). While observing consecutive cuts, regardless of NS, it can be noticed that the highest values for TPC, TNFC, and TFC were achieved at C II. Cutting and damaging plant tissue during harvest can be considered a form of stress whereby the plant needs to heal wounds and regrow. Numerous research (Hu et al., 2022; Pratyusha, 2022; Dujmović et al., 2023) confirm that various stress conditions stimulate phenols production in plant organisms which may explain the increased phenolic content in C II.

As established, N availability can influence the content of various phenolic compounds in plants through several mechanisms, but data on the influence of NSs with different N content on individual phenolic compounds are lacking. The significance of the factor interactions (NS × C) shows that the combination of both factors strongly affected individual phenolic content (**Table 7**). The results of the present study correspond with some previous literature data. The presence of caffeic, chlorogenic, coumaric, and ferulic acid, kaempferol and quercetin 3-glucoside in nettle was detected in the study by Repajić et al. (2021). In another research, carried out by Dujmović et al. (2023), a similar content of coumaric, ellagic, and ferulic acid, as well as naringin was identified in nettle leaves as in our results. Caffeoylmalic acid was the most abundant and chlorogenic acid was the 2^nd^ most represented phenolic compound in nettle in the work of Koczkodaj et al. (2023), and the same trend was observed in the results of the present study. Different levels of N can cause an increase or decrease in different phenolic compounds, which was the case in this study. Obtained results show that the lowest N content (NS1) at the C II was most suited for increased accumulation of caffeic, caffeoylmalic, and ferulic acid and naringin (10.32, 462.38, 24.02, and 16.55 mg/L respectively), while quite the opposite was in the case of chlorogenic acid where the highest N levels (NS4) favored the increase of chlorogenic acid content (119.57 mg/L).

**Table 7:** Individual phenolic compound content of nettle cultivated in different nutrient solutions.

Treatment	Caffeic acid	Caffeoylmalic acid	Chlorogenic acid	Coumaric acid	Ellagic acid	Ferulic acid	Kaempferol	Naringin	Quercetin 3 glucoside	Rutin trihydrate
mg/L										
NS1 × C I	nd	9.14^e^ ± 1.79	10.68^e^ ± 1.91	nd	0.58^c^ ± 0.08	3.68^c^ ± 0.09	11.91^d^ ± 0.01	8.35^cd^ ± 0.23	7.01^c^ ± 0.05	7.37^c^ ± 0.08
NS2 × C I	0.11^d^ ± 0.11	25.29^e^ ± 7.28	20.26^e^ ± 5.21	0.07^c^ ± 0.07	0.70^c^ ± 0.09	3.88^c^ ± 0.14	11.95^cd^ ± 0.03	8.48^bcd^ ± 0.26	7.12^c^ ± 0.07	7.49^c^ ± 0.09
NS3 × C I	0.28^d^ ± 0.19	46.28^e^ ± 1.08	8.33^e^ ± 0.62	nd	1.83^c^ ± 0.06	4.82^c^ ± 0.11	11.94^cd^ ± 0.01	9.10^bc^ ± 0.09	7.41^c^ ± 0.18	8.55^c^ ± 0.05
NS4 × C I	nd	10.12^e^ ± 0.71	10.67^e^ ± 1.21	nd	0.91^c^ ± 0.12	4.03^c^ ± 0.04	11.98^bcd^ ± 0.04	8.14^d^ ± 0.13	7.18^c^ ± 0.04	7.68^c^ ± 0.14
NS1 × C II	10.32^a^ ± 0.12	462.38^a^ ± 23.12	104.00^b^ ± 15.12	5.27^b^ ± 0.95	14.40^a^ ± 1.34	24.02^a^ ± 3.77	12.03^abc^ ± 0.10	16.55^a^ ± 0.73	14.14^ab^ ± 0.23	24.00^a^ ± 2.40
NS2 × C II	4.41^c^ ± 0.14	269.34^c^ ± 13.47	48.40^d^ ± 0.90	4.63^b^ ± 1.74	12.70^ab^ ± 2.55	15.98^b^ ± 3.23	12.00^bcd^ ± 0.06	8.59^bcd^ ± 0.19	13.84^b^ ± 1.62	21.92^a^ ± 5.07
NS3 × C II	6.77^b^ ± 0.17	229.08^d^ ± 26.60	69.07^c^ ± 3.27	9.72^a^ ± 0.12	0.48^c^ ± 0.00	18.30^b^ ± 1.00	12.13^a^ ± 0.01	7.97^d^ ± 0.08	14.97^ab^ ± 0.54	13.94^b^ ± 1.00
NS4 × C II	7.00^b^ ± 0.09	365.80^b^ ± 16.48	119.57^a^ ± 3.93	10.52^a^ ± 0.75	10.34^b^ ± 0.52	19.34^b^ ± 0.46	12.09^ab^ ± 0.02	9.21^b^ ± 0.16	15.54^a^ ± 0.25	19.27^ab^ ± 1.53
ANOVA	p ≤ 0.0001	p ≤ 0.0001	p ≤ 0.0001	p ≤ 0.0001	p ≤ 0.0001	p ≤ 0.0001	p ≤ 0.0019	p ≤ 0.0001	p ≤ 0.0001	p ≤ 0.0001
NS	0.0001^***^	0.0001^***^	0.0001^***^	0.0001^***^	0.0001^***^	0.0192^**^	0.0377^**^	0.0001^***^	0.0686^ns^	0.0158^*^
C	0.0001^***^	0.0001^***^	0.0001^***^	0.0001^***^	0.0001^***^	0.0001^***^	0.0001^***^	0.0001^***^	0.0001^***^	0.0001^***^
NS × C	0.0001^***^	0.0001^***^	0.0001^***^	0.0001^***^	0.0001^***^	0.0001^***^	0.0019^**^	0.0001^***^	0.0001^***^	0.0001^***^

NS, nutrient solution; C, consecutive cut; NS × C, the interaction of nutrient solution and consecutive cut; C I, 1^st^ cut; C II, 2^nd^ cut. Results are expressed as mean ± standard deviation. Different letters indicate significant differences between mean values. nd – not determined; ns, *, **, and *** indicate non-significant and significant effects at p < 0.05, 0.01, and 0.001, respectively.

Grevsen et al. (2008) discovered that the application of different N levels to nettle either had no influence on caffeoylmalic acid and quercetin 3-glucoside, or caused their increase when lowering content of N which can be related to the results of this research. A large influence of cut as a stress factor can be observed in the case of individual phenolic compounds. All phenolics investigated in this study show the highest values in the C II, assuming that their enhanced synthesis helps the plant cope more easily with tissue damage. In fact, the average value of caffeoylmalic acid increased even over 1300% from the C I to the C II, while the content of chlorogenic acid increased by about 580% in the C II. In addition, the presence and functions of different phenols are highly influenced by environmental factors (Šamec et al., 2021), so the abiotic conditions with an average air temperature of 25°C, and RH of 24% during the C II might also favor phenolic accumulation. Regarding total and individual phenolic content, it can be summarized that a balanced and appropriate N supply is crucial to optimize the content of phenolic compounds, but excessive N levels can sometimes lead to a decrease in certain phenolics, due to an overproduction of vegetative growth, as confirmed by the results of the present study.

### 3.7 Antioxidant activity of nettle

The accumulation of antioxidants, and thus the antioxidant capacity of plants can be influenced by a variety of factors, including environmental factors and nutrient availability (Liu et al., 2016; Weiwei et al., 2020; Villani et al., 2023), and according to Weiwei et al. (2020), N availability plays an essential role in regulating antioxidant accumulation. Considering the analyzed antioxidant capacity of nettle leaves grown under different treatments, it should be emphasized that the two varied factors (NS and C) and their interaction (NS × C) significantly affected antioxidant capacity. The results for the FRAP assay ranged from 963.27 to 3237.62 µmol TE/L and varied significantly, while the results for ABTS assay ranged from 2365.24 to 2525.78 µmol TE/L, as presented in **Table 6**. Since all major antioxidants (AsA and phenolic compounds) showed the highest content in the NS2 × C II treatment, it was expected that the highest antioxidant capacity would be detected in the same treatment. The results of our research show that lower N doses in the nutrient solution (NS1 and NS2) promoted antioxidant capacity, which can be related to the study of Olarewaju et al. (2018), which found that the antioxidant capacity of some leafy vegetables depended on N fertilizer dose. However, the data on antioxidant capacity from different studies are contradictory showing a trend of improving (Ma et al., 2023) or lowering (Ibrahim et al., 2013) bioactive content and antioxidant capacity of plant material by increasing the content of nitrogen in fertilizer. On the other hand, the influence of stress caused by cutting is pronounced in the results obtained by the FRAP method, while it is not noticeable in the case of the ABTS assay. These two methods were used because phenols are a mixture of hydrophilic and lipophilic compounds, which is why the results of antioxidant capacity obtained by both methods are different. But regardless of the different results, this research shows that the nettle leaves have a strong antioxidant capacity, which is consistent with other studies (Radman et al., 2015; Dujmović et al., 2023). The obtained results also show that the antioxidant capacity of nettle leaves is strongly influenced by the composition of the NS and consecutive cuts, whereby the proper selection of these factors can increase the antioxidant activity of nettle.

### 3.8 Photosynthetic pigment content of nettle

In general, photosynthetic pigments, including chlorophyll a (Chl_a), chlorophyll b (Chl_b), and total chlorophylls content (TCh), were higher in C I compared to the C II, with Chl_a content 21% higher, Chl_b content 17% higher, and TCh content 19% higher (**Table 8**). But the results of total carotenoids content (TCa) do not coincide with the trend recorded for chlorophylls depending on the consecutive cut, during which about 19% higher values were recorded in nettle leaves in C II compared to the C I. Given the results of significance of each varied factor, cut significantly affected the content of all analyzed photosynthetic pigments. In C I the highest TCh value was recorded in leaves treated with NS3 and NS4 with no significant statistical difference, while in C II in leaves treated by NS1 and NS4 with no significant difference. Content of TCA in C I was the highest in nettle leaves treated by NS1 and NS3 with no significant difference, while in C II in leaves treated by NS1 and NS4 with no significant difference. According to the results of the significance of influence of individual varied factors, nutrient solution content (NS) had no significant influence on analyzed photosynthetic pigments content, while the combination of both factors NS × C significantly influenced only on the TCA content.

**Table 8:** Pigment compounds content of nettle cultivated in different nutrient solutions.

Treatment	Chl_a	Chl_b	TCh	TCa
	μg/g			
NS1 × CI	0.73^ab^ ± 0.02	0.39^ab^ ± 0.01	1.11^ab^ ± 0.01	0.21^ab^ ± 0.01
NS2 × CI	0.77^a^ ± 0.01	0.39^ab^ ± 0.03	1.15^ab^ ± 0.04	0.20^b^ ± 0.02
NS3 × CI	0.78^a^ ± 0.11	0.42^a^ ± 0.09	1.20^a^ ± 0.20	0.21^ab^ ± 0.01
NS4 × CI	0.77^a^ ± 0.03	0.44^a^ ± 0.03	1.21^a^ ± 0.00	0.15^c^ ± 0.03
NS1 × CII	0.67^ab^ ± 0.05	0.40^ab^ ± 0.03	1.07^abc^ ± 0.08	0.24^a^ ± 0.01
NS2 × CII	0.61^bc^ ± 0.05	0.35^ab^ ± 0.06	0.95^bc^ ± 0.10	0.21^ab^ ± 0.01
NS3 × CII	0.56^c^ ± 0.04	0.30^b^ ± 0.02	0.86^c^ ± 0.06	0.22^ab^ ± 0.01
NS4 × CII	0.69^abc^ ± 0.10	0.34^ab^ ± 0.05	1.03^abc^ ± 0.15	0.24^a^ ± 0.03
ANOVA	p ≤ 0.0079	p ≤ 0.0214	p ≤ 0.0096	p ≤ 0.0011
NS	0.4212^ns^	0.5959^ns^	0.4505^ns^	0.0936^ns^
C	0.0001^***^	0.0064^**^	0.0004^***^	0.0001^***^
NS × C	0.0079^**^	0.0214^*^	0.0096^**^	0.0011^***^

NS, nutrient solution; C, consecutive cut; NS × C, the interaction of nutrient solution and consecutive cut; C I, 1^st^ cut; C II, 2^nd^ cut.; Chl_a. chlorophyll a content; Chl_b. chlorophyll b content; TCh, total chlorophyll content; TCa, total carotenoid content. Results are expressed as mean ± standard deviation. Different letters indicate significant differences between mean values. ns, *, **, and *** indicate non-significant and significant effects at p < 0.05, 0.01, and 0.001, respectively.

As research studies suggest, chlorophyll and carotenoid content in plants is strongly influenced by ecological factors, especially temperature and solar radiation (Kukrić et al., 2012; Kriegel et al., 2018; Paulauskienė et al., 2021), but also by harvest time and the phenophase of the plant (Kukrić et al., 2012; Šircelj et al., 2018). Photosynthetic pigments tend to accumulate in young leaves, while the concentration of these pigments decreases during senescence and plant ageing (Biesiada et al., 2010; Kukrić et al., 2012; Wujeska-Klause et al., 2019). This statement is in agreement with the results of chlorophyll content in nettle leaves from this study, as the values of Chl_a, Chl_b, and TCh are significantly higher after C I and significantly lower after C II. TCA values do not follow the same trend as chlorophyll pigments when considering the consecutive cuts, as the generally higher TCA levels were found in C II. This could be influenced by the more intense sunlight in July, when the 2^nd^ cut was carried out, as other authors have also found the higher TCA content in leaves in places with greater availability of sunlight (Paulauskienė et al., 2021). In addition, as vital components of the plant photosynthetic process, carotenoids function as light scavengers, energy carriers, and photochemical redox reactants that can perform a photoprotective function when plants are to exposed to sunlight (Shonte et al., 2020). Furthermore, strong link between photosynthetic pigments and nitrogen have been proven by several research studies (Chen et al., 2018; Botelho and Müller, 2020; Peng et al., 2021). Nitrogen is an essential component of the chlorophyll molecule and plays a crucial role in the most important plant metabolic processes, photosynthesis and the biosynthesis of chlorophylls. Thus, a deficiency of nitrogen leads to a decrease in the content of chlorophylls and carotenoids due to the reduced leaf CO_2_ assimilation (Lin et al., 2016). Considering the above, the results of higher photosynthetic pigment content in treatments with higher nitrogen content in the nutrient solutions (NS3 and NS4) are consistent with the data from the literature (Huang et al., 2021). This trend is more pronounced in C I, where Chl_a, Chl_b and TCh levels were significantly higher in treatments with NS3 and NS4, while in C II some deviations were recorded, as the highest chlorophyll levels were recorded in treatments with NS1 and NS4. This could be due to a stronger effect of the consecutive cut i.e. plant age on the content of photosynthetic pigments. Generally, given the other literature data about chlorophyll and carotenoid content in nettle leaves can be pointed that results from this study are in agreement with other researches which state that nettle is a rich source of chlorophylls. In this research even higher values of photosynthetic pigments were recorded compared to the other literature data (Guil-Guerrero et al., 2003; Kukrić et al., 2012; Kriegel et al., 2018; Paulauskienė et al., 2021; Opačić et al., 2022).

## 4 Conclusion

The research was conducted in a greenhouse hydroponic system and offers insights into controlled agronomic practices for nettle cultivation. These results are promising for sustainable agriculture in the face of growing environmental challenges. They demonstrate the potential of hydroponic methods for cultivation of nettle as a green leafy vegetable for human consumption and optimize resource use and food quality. It can be concluded that hydroponically grown nettle has high nutritional quality and is an excellent source of minerals and specialized metabolites that enrich human nutrition. Considering the results of this study, a nutrient solution with lower nutrient levels (NS2) yielded the plant material with the highest content of ascorbic acid, total phenolic content, total non-flavonoid content, and total flavonoid content, as well as antioxidant capacity during 2^nd^ consecutive cut. Nutrient solution with even lower levels of nutrients (NS1) resulted in the lowest nitrate content of the plant material (which is critical for both safety and nutritional value) and the highest levels of Ca and Fe, as a result of ion antagonism. Additionally, NS1 resulted in higher concentrations of specific phenolic compounds: caffeoyl maleic acid, ellagic acid, ferulic acid, naringin, and rutin trihydrate. Based on all these points, and considering that this solution is the most favorable in terms of resource management (benefits in terms of cost savings, reduced environmental impact and efficient use of resources) due to the lower content of nutrients, we would highly recommend it for the future sustainable cultivation of nettle using hydroponic techniques.

## Data availability statement

The raw data supporting the conclusions of this article will be made available by the authors, without undue reservation.

## Author contributions

NO: Conceptualization, Data curation, Software, Writing – original draft, Writing – review & editing. SR: Conceptualization, Methodology, Writing – original draft, Writing – review & editing. MD: Conceptualization, Data curation, Methodology, Writing – original draft, Writing – review & editing. SFU: Methodology, Software, Writing – review & editing. BB: Software, Writing – review & editing. NT: Software, Visualization, Writing – review & editing. MP: Conceptualization, Data curation, Writing – original draft. LČ: Data curation, Writing – review & editing. SV: Data curation, Visualization, Writing – review & editing. JŠŽ: Conceptualization, Methodology, Writing – original draft, Writing – review & editing.
